# Synthesis of three-dimensional hierarchical furball-like tungsten trioxide microspheres for high performance supercapacitor electrodes

**DOI:** 10.1039/c9ra10995a

**Published:** 2020-04-01

**Authors:** Xu He, Xiangyue Wang, Bangning Sun, Junning Wan, Yu Wang, Dong He, Hui Suo, Chun Zhao

**Affiliations:** State Key Laboratory of Integrated Optoelectronics, College of Electronic Science and Engineering, Jilin University Changchun 130118 China hedong@jlu.edu.cn zchun@jlu.edu.cn; College of Chemistry, Jilin University Changchun 130118 China

## Abstract

A hierarchical furball-like WO_3_ electrode material, based on stainless-steel mesh, was successfully synthesized *via* a simple *in situ* hydrothermal method. The electrode materials obtained are made from a self-assembled nanorod core and a connected/quasi-connected nano-thorn network shell, and could help utilize all the surface or near-surface regions for faradaic reaction. Furthermore, the furball-like WO_3_ special microstructure provides a more effective charge storage area, exhibiting a high specific capacitance of 8.35 F cm^−2^ and excellent cycling stability (93.4% of its initial value after 10 000 cycles). These performances indicate this furball-like WO_3_ material would be a promising candidate for high performance supercapacitors.

## Abstract 2

Furball-like tungsten trioxide microspheres with significant specific capacitance and remarkable cycle stability.

## Graphical abstract

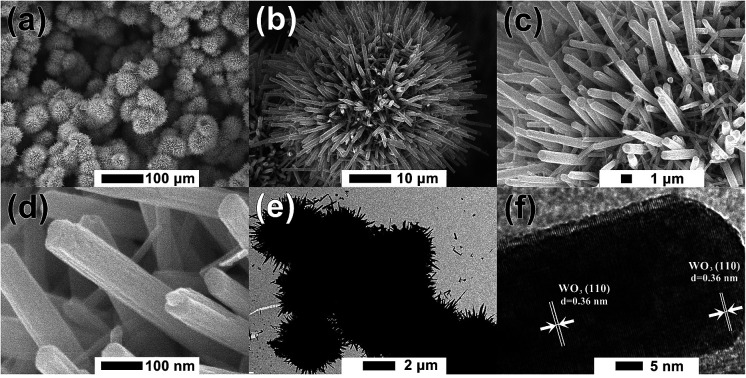

## 1. Introduction

With the advantages of high-power density, long cycling stability, high conversion efficiency and easy maintenance, supercapacitors have become a promising candidate for renewable energy conversion and storage devices, especially in photovoltaics and wind power generation and transition.^1,2^ But their application in renewable energy field is limited by their relatively low specific capacitance and relatively high average equivalent series resistance caused by the preparation technique.^3^ According to the energy storage mechanism, supercapacitor electrode materials can be classified into two types, one is electrical double-layer capacitive material and another one is pseudocapacitor material. It is well known that pseudocapacitive materials can obtain higher specific capacitance than double-layer capacitive materials by their unique energy storage method, which depends on the Faraday reactions on the surface or near-surface regions of the microstructure of the materials to charge and discharge. Thus, methods to control the structures of pseudocapacitive materials (*i.e.*, morphology and crystal structure) should be developed to improve the charge and ion-transfer efficiency and to enhance the utilization of the pseudoactive material. To design and synthesize high performance transition metal oxides electrode materials with various nano/micro morphologies and spatial structures can significantly improve the electrochemical performance of the supercapacitors.^4,5^ For example, Sun *et al.*^6^ designed a flower-like NiO/PANI material electrode with a developed specific capacitance of 2565 F g^−1^ and excellent cycling stability. Huang *et al.*^7^ designed hollow and spinous NiCo_2_S_4_ nanotubes template by natural silk exhibiting a better electrochemical performance than the solely vulcanized samples. Zhang *et al.*^8^ designed and prepared NiO@N–C electrode with double-shell hollow structures that demonstrated a specific capacitance of 782 F g^−1^. However, the structure of active material would not remain same to the structure of electrode material after crushing method. It is believed that, with a more stable structure and stronger bonding with current collector, the electrode prepared *via in situ* method can gain a better cycle life and shelf-life.

In recent years, the tungsten oxides have been researched as supercapacitor electrodes considering their multiple oxidation states, high electronic conductivity and fast ion insertion/deinsertion. Ibrahim *et al.*^9^ prepared WO_3_-rGO nanocomposite, using a simple method of pulsed laser ablation in liquids, and revealed a specific capacitance of 577 F g^−1^. Xu *et al.*^10^ synthesized WO_3_ mesoscopic microspheres self-assembled by nanofibers that exhibited a specific capacitance of 797.05 F g^−1^. Though remarkable progresses have been achieved, the transition metal oxides electrode materials are still limited by the relatively short cycle life caused by the expansion/contraction during charging and discharging process.^11,12^ To address this challenge, *in situ* method was widely utilized to strength the bonding between active material and the current collector. Furthermore, fabricating of 3D porous structures instead of bulks structures can also be of great help to high cycle stability.

In this study, the hierarchical 3D furball-like WO_3_ microspheres with the self-assembled nanorods core and the connected (and quasi-connected) nano-thorns network shell have been synthesized by hydrothermal method. The unique microstructures can minimize the structural damage during the charging and discharging process and provide numerous active sides for the faradaic reactions, which can increase the utilization of pseudocapacitive materials. Furthermore, with the help of *in situ* method, this WO_3_ material have a stronger bonding with current collector. Hence, the prepared WO_3_ electrode exhibited a high specific capacitance, low average equivalent series resistance, and excellent stability, demonstrating a great potential in practical application.

## 2. Materials and methods

All reagents used in the experiment were purchased from Sinopharm Chemical Reagent Co. Ltd. The stainless-steel mesh (SSM) (purchased from ToughMan Co. Ltd) was trimmed into 1 × 1.5 cm^2^ pieces, washed by toluene, acetone, ethanol and deionized water in sequence under ultrasonic condition, and finally dried in an electric oven under 40 °C for 3 h. The 3D furball-like WO_3_ microspheres were prepared by a one-step hydrothermal method following by an annealing process. In brief, 10.6 mL of 3 M HCl solution was dropwise added into 100 mL 0.125 M of Na_2_WO_3_·2H_2_O aqueous solution under vigorous magnetic stirring. Then, the previous solution was mixed with 100 mL of 0.3125 M H_2_C_2_O_4_ solution with magnetic stirring for 10 min. The mixture solution was transferred to a volumetric flask and diluted to 250 mL with deionized water and allow the solution to stand for at 1 h. Subsequently, 30 mL of previous solution was transferred into a Teflon-lined stainless autoclave and 9.375 mmol of (NH_4_)_2_SO_4_ was added into the solution under magnetic stir for 30 min. Then, two pieces of pre-washed SSM slices were put into the autoclave. Then, the autoclave was sealed and maintained at 180 °C for 16 h. After the autoclave cooled down to room temperature naturally, the samples were rinsed thoroughly with deionized water and ethanol. Finally, the samples were annealed in a muffle furnace at 450 °C for 16 h. The active materials on the SSM surface is about 11.8 mg cm^−2^.

### Methods and characterization

The micro/nano morphologies and structures were obtained by scanning electron microscopy (SEM) images on a JEOL JEM-6700F instrument and the transmission electron microscopic (TEM) images and high-resolution transmission electron microscopic (HRTEM) images on a JEOL JEM-2100F instrument with an acceleration voltage of 200 kV. The crystal composition and phase of the prepared samples were evaluated by X-ray diffraction (XRD) method on a Shimadzu 6000 instrument with Cu Kα radiation (*λ* = 1.54056 Å) and the X-ray photoelectron spectroscopy (XPS) method on an ESCALAB 250 instrument.

### Electrochemical measurements

All the electrochemical measurements including cyclic voltammetry (CV), galvanostatic charge–discharge (GCD) and cycling stability tests were conducted on a Chenhua CHI660D electrochemical workstation in a three-electrode cell using a platinum electrode (1.5 × 1.5 cm^2^) as the counter electrode, a SCE electrode as the reference electrode, the prepared furball-like WO_3_ electrode sample as working electrode, and 2 M H_2_SO_4_ aqueous solution as electrolyte.

## 3. Results and discussion

**Fig. 1:**
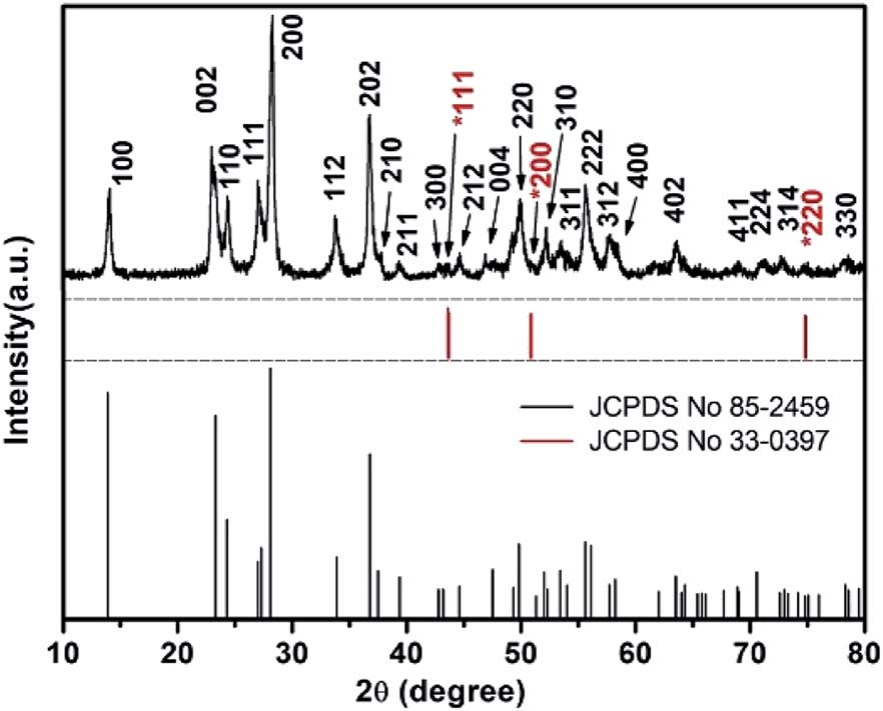
The XRD pattern of the furball-like WO_3_ microspheres sample.

Fig. 1 shows the XRD patterns of furball-like WO_3_ sample. The peaks marked with asterisks are derived from the SSM substrate (JCPDS No. 033-0397) and all other peaks can be indexed to (100), (002), (110), (111), (200), (112), (202), (210), (211), (300), (212), (004), (220), (310), (311), (222), (312), (400), (402), (411), (224), (314), and (330) planes of the hexagonal WO_3_ (JCPDS no. 085-2459), with lattice constants *a* = 7.332 Å, *c* = 7.663 Å, where no peaks of impurities can be detected, revealing the high purity and high crystallinity of the sample.

**Fig. 2:**
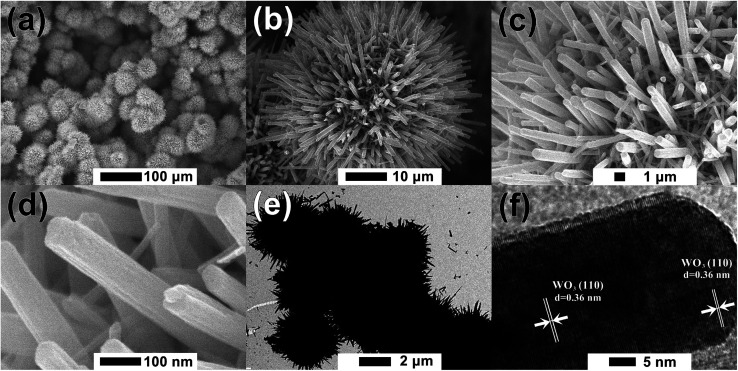
(a)–(d) Low to high magnification SEM, (e) TEM and (f) HRTEM images of the furball-like WO_3_ microspheres sample.

The surface morphologies of the furball-like WO_3_ microspheres on the SSM substrate were characterized through SEM, TEM and HRTEM methods as shown in Fig. 2. Fig. 2(a) shows that the surface of the SSM is fully covered by furball-like WO_3_ microspheres. The diameters of the WO_3_ microspheres are in a range of about 3 to 5 μm (Fig. 2(b)) and the microspheres connected and quash-connected to each other with the fluffy nanorods forming a first level interconnected hierarchically porous structure network. This 3D network can provide more active site for Faraday reaction and largely get rid of structural damage during the charging/discharging process. Hence, this furball-like structure can help developing electrochemical performance as well as improving the cycle stability of the electrode. Typical low and high magnification images of furball-like WO_3_ microsphere are shown in Fig. 2(c) and (d). The WO_3_ microspheres are assembled by numerous WO_3_ nanorods with diameters of 20 to 110 nm and length of 500 to 1100 nm forming a furball-like structure which is also confirmed by the TEM image in Fig. 2(e). These WO_3_ nanorods assembled microspheres exhibit a second level interconnected porous structure network with nano/micro pores with different sizes. As shown in the HRTEM image, the interlayer spacing distance of the crystalline WO_3_ nanorods is measured to be 0.36 nm, which corresponds to the (110) crystal plane of WO_3_. The unique binder-free first and the second porous structure network on the SSM substrate can provide significantly amount active sites for redox reactions, provide the buffer space during the ions insertion/desertion processes minimize effect of the structure failure, and provide strong adhesive between the electroclinical active material and the substrate.

**Fig. 3:**
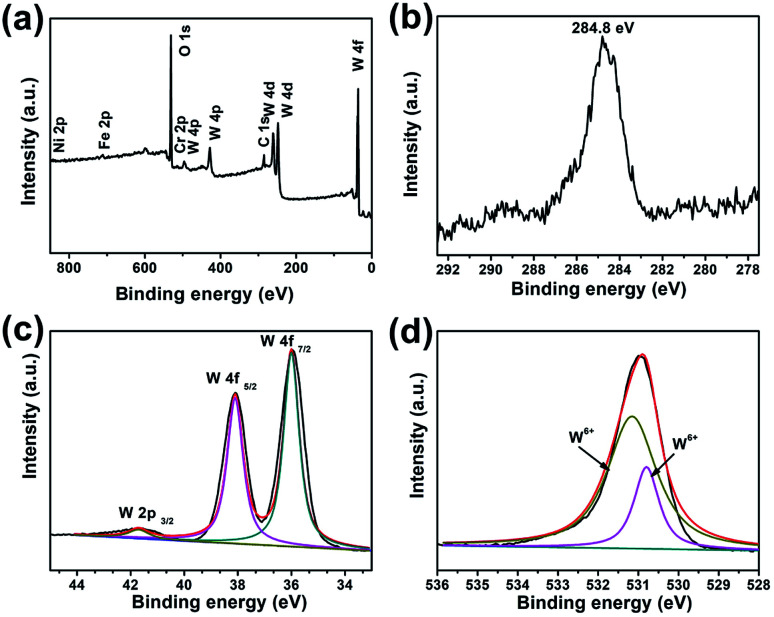
XPS spectra of furball-like WO_3_ microspheres sample: (a) survey XPS spectrum, (b) core-level XPS spectra of (b) C 1s, (c) W 4f, and (d) O 1s.

X-ray photoelectron spectroscopy (XPS) measurements were performed to further identify the chemical composition and the valence states of the furball-like WO_3_ microspheres sample. The results were shown in Fig. 3. All the spectra were corrected for specimen charging by referencing the C 1s to 284.8 eV (Fig. 3(b)). The complete spectrum of furball-like WO_3_ sample indicates the presence of tungsten, nickel, iron, chromium, carbon and oxygen elements and no other impurities can be detected. The nickel, iron and chromium elements were from the SS substrate which was also corresponding to the XRD results. Fig. 3(c) shows the high-resolution spectrum of W 4f core level of the furball-like WO_3_ sample, and the main peaks located at 36 and 38.1 eV can be assigned to the binding energy of W 4f_7/2_ and W 4f_5/2_ with a spin-energy separation of 2.1 eV, indicating the valence of the W element is W^6+^ in the sample.^13^ At the same time, one small lonely peak representing the W 2p_3/2_ can be found at 41.7 eV, which also reveals the existence of W^6+^ states.^14,15^ The XPS spectrum of O 1s is shown in Fig. 3(d), it exhibits that the detected peak can be fitted into two peaks with binding energies at 530.8 and 531.0 eV which can be indexed to the metal–oxygen bonds of the WO_3_ phase. These results together with the XRD data further confirmed the chemical composition and the purity of the furball-like WO_3_ microspheres sample.

**Fig. 4:**
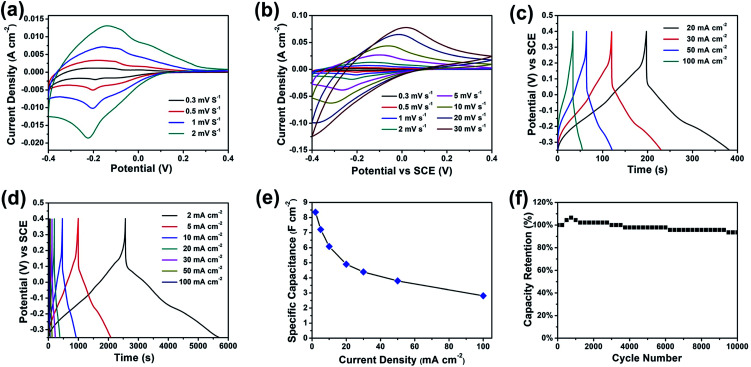
(a and b) CV curves, (c and d) GCD curves, (e) specific capacitance, and (f) cycling performance of the furball-like WO_3_ microspheres electrodes.

The electrochemical performance of the furball-like WO_3_ was evaluated in a conventional three-electrode configuration in 2 M H_2_SO_4_ aqueous solution by cyclic voltammetry (CV) and galvanostatic charge–discharge (GCD) measurements. Fig. 4(a) and (b) show the CV curves of the furball-like WO_3_ electrode from low to high scan rates at the potential widow from −0.4 to 0.4 V. All the profiles of the CV curves exhibit non-rectangular shape, revealing the electron charge/discharge process of the WO_3_ electrode was dominated by pseudocapacitive capacitance characteristics. As shown in Fig. 4(a), one obvious pair of anodic and cathodic peak can be found at the lower scan rates, and the anodic and cathodic peaks begin to shift towards to positive and negative potential with the increasing of the scan rate Fig. 4(b), while the peak current density can reach to 13 mA cm^−2^ even at a low scan rate of 2 mV s^−1^ and a high current density of 750 mA cm^−2^ at a scan rate of 30 mV s^−1^, revealing the good reversibility as well as the fast charge transfer and ions insertion/desertion properties of this material.

The properties of the WO_3_ pseudocapacitance can be explained by the following equation:^16^

1 WO_3_ + *x*H^+^ + *x*e^−^ ↔ H_*x*_WO_3_

Fig. 4(c) and (d) show the galvanostatic charge–discharge (GCD) curves of the furball-like WO_3_ at different current densities. The profiles of the GCD curves reveal quash linear characteristics and one slight curvature change at about −0.2 V which is consisted with the CV curves indicating the faradaic pseudocapacitive characteristic of the WO_3_ electrode.

The specific capacitance calculated based on the GCD curves is shown in Fig. 4(e) according to the following equations:^17^2
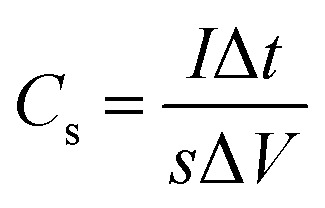
3
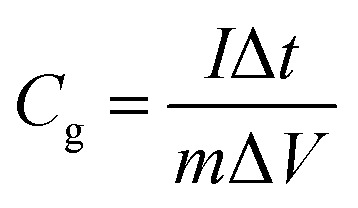
where *C*_s_ (F cm^−2^) and *C*_g_ (F g^−1^) are the specific capacitances, *I* (A) is the charge–discharge current, Δ*t* (s) is the discharging time, Δ*V* (V) is the voltage window, *s* (cm^2^) is the effective area of the electrode and *m* (g) is the mass of the active material within the electrode. The specific capacitances (*C*_s_) are calculated to be 8.35, 7.21, 6.08, 4.90, 4.39, 3.8 and 2.81 F cm^−2^ at 2, 5, 10, 20, 30, 50 and 100 mA cm^−2^, respectively (Fig. 4(e)). And the gravimetric specific capacitance (*C*_g_) can be calculated to 708.0, 610.7, 515.5, 415.4, 372.2, 322.0, and 238.4 F g^−1^ at 2, 5, 10, 20, 30, 50 and 100 mA cm^−2^, respectively. Fig. 4(f) shows the excellent cycling stability of the WO_3_ electrode. The capacity retention slightly increases in the first 800 cycles, which may cause by the electrolyte penetration through the WO_3_ networks and the activation of the electrode. Compared with other literatures, this furball-like WO_3_ material has a better performance in both working potential and specific capacitance as shown in Table 1. Then the retention slightly decreases to 93.4% of its initial value after 10 000 cycles, which shows a better cycling performance than previously reports,^18–20^ indicating the excellent cycling stability and the practical application potential of the furball-like WO_3_ electrode.

**Table 1:** Comparison of the specific capacitance for furball-like WO_3_ with the literature

Active material	Collector	Working voltage	Current density	Specific capacitance	Ref.
MWCNT-WO_3_	Carbon cloth	−0.6 to 0 V	2 mA cm^−2^	1.55 F cm^−2^	19
WO_3_ microspheres	Stainless steel	−0.45 to 0 V	0.5 mA cm^−2^	0.751 F cm^−2^	5
h-WO_3_ nanorod	Carbon cloth	−0.7 to 0 V	4 mA cm^−2^	0.377 F cm^−2^	12
WO_3_@PPy	Stainless steel	−0.8 to 0 V	2 A g^−1^	586 F g^−1^	2
Pd–WO_3_	Glassy carbon electrode	−0.7 to 0.1 V	0.5 A g^−1^	33.34 F g^−1^	3
Furball-like WO_3_	Stainless steel	−0.3 to 0.4 V	2 mA cm^−2^	8.35 F cm^−2^ (708 F g^−1^)	This work

## 4. Conclusions

In summary, the high-performance 3D furball-like SS@WO_3_ electrodes have been synthesized by hydrothermal method, which can be used as a binder-free electrode without further processing for supercapacitors. The synergetic contribution from the 3D furball-like WO_3_ microstructures which forming the unique connected and quasi-connected WO_3_ networks and the SS substrate accounts for the high specific capacitance of 8.35 F cm^−2^ and excellent cycling performance. These results indicate the furball-like SS@WO_3_ electrode would be a most promising one for supercapacitor applications and also proved the SS@WO_3_ electrode demonstrates a good potential for environmental benign, cost-effective and high performance energy storage devices.

## Conflicts of interest

There are no conflicts to declare.

## Supplementary Material
